# Diffusion Tensor Imaging of Parkinson’s Disease, Multiple System Atrophy and Progressive Supranuclear Palsy: A Tract-Based Spatial Statistics Study

**DOI:** 10.1371/journal.pone.0112638

**Published:** 2014-11-18

**Authors:** Amanda Worker, Camilla Blain, Jozef Jarosz, K. Ray Chaudhuri, Gareth J. Barker, Steve C. R. Williams, Richard G. Brown, P. Nigel Leigh, Flavio Dell’Acqua, Andrew Simmons

**Affiliations:** 1 Institute of Psychiatry, King’s College London, London, United Kingdom; 2 National Institute for Health Research Biomedical Research Centre for Mental Health at South London and Maudsley NHS Foundation Trust and Institute of Psychiatry, King’s College London, London, United Kingdom; 3 National Institute for Health Research Biomedical Research Unit for Dementia at South London and Maudsley NHS Foundation Trust and Institute of Psychiatry, King’s College London, London, United Kingdom; 4 King’s College Hospital, London, United Kingdom; 5 Trafford Centre for Biomedical Research, Brighton and Sussex Medical School, University of Sussex, Falmer, Brighton, United Kingdom; University of Ulm, Germany

## Abstract

Although often clinically indistinguishable in the early stages, Parkinson’s disease (PD), Multiple System Atrophy (MSA) and Progressive Supranuclear Palsy (PSP) have distinct neuropathological changes. The aim of the current study was to identify white matter tract neurodegeneration characteristic of each of the three syndromes. Tract-based spatial statistics (TBSS) was used to perform a whole-brain automated analysis of diffusion tensor imaging (DTI) data to compare differences in fractional anisotropy (FA) and mean diffusivity (MD) between the three clinical groups and healthy control subjects. Further analyses were conducted to assess the relationship between these putative indices of white matter microstructure and clinical measures of disease severity and symptoms. In PSP, relative to controls, changes in DTI indices consistent with white matter tract degeneration were identified in the corpus callosum, corona radiata, corticospinal tract, superior longitudinal fasciculus, anterior thalamic radiation, superior cerebellar peduncle, medial lemniscus, retrolenticular and anterior limb of the internal capsule, cerebral peduncle and external capsule bilaterally, as well as the left posterior limb of the internal capsule and the right posterior thalamic radiation. MSA patients also displayed differences in the body of the corpus callosum corticospinal tract, cerebellar peduncle, medial lemniscus, anterior and superior corona radiata, posterior limb of the internal capsule external capsule and cerebral peduncle bilaterally, as well as the left anterior limb of the internal capsule and the left anterior thalamic radiation. No significant white matter abnormalities were observed in the PD group. Across groups, MD correlated positively with disease severity in all major white matter tracts. These results show widespread changes in white matter tracts in both PSP and MSA patients, even at a mid-point in the disease process, which are not found in patients with PD.

## Introduction

Parkinson’s disease (PD), Multiple System Atrophy (MSA) and Progressive Supranuclear Palsy (PSP) are neurodegenerative diseases that are characterized by very similar motor symptoms, making them difficult to distinguish in the early stages [1], [2], despite having distinct molecular pathology [3]–[5].

A range of Magnetic Resonance Imaging (MRI) techniques have been used to identify regions of brain pathology in parkinsonian syndromes. Previous imaging studies have highlighted pathological changes in white matter, with some involvement of the cortex [6]–[10]. Diffusion tensor imaging (DTI) provides an indirect insight into white matter microstructure in disease [11]. Frequently used DTI measures include fractional anisotropy (FA) (directionality of water molecule movement within white matter fibers and axons) and mean diffusivity (MD) (degree to which water molecules move within tissues). Studies utilising FA, MD and other diffusion measures in PSP have described abnormalities in the superior longitudinal fasciculus, corpus callosum [12], [13] and superior cerebellar peduncles [13]–[17], whilst in MSA white matter abnormalities have been identified in the putamen [8] and middle cerebellar peduncles [18], [19]. In non-demented PD, white matter is thought to remain largely normal [20], however there have been reports of corpus callosum, superior cerebellar peduncles, cingulum and uncinate involvement [17].

Previous neuroimaging studies have often taken a Region of Interest (ROI) approach and few DTI studies have been carried out to characterize the whole brain white matter pathology of patients with Parkinson’s Plus syndromes. Tract-Based Spatial Statistics (TBSS) is a whole-brain skeleton-based technique [21] that enables differences in measures such as FA and MD, that may reflect the microstructural properties of white matter tracts, to be identified, whilst avoiding the need for a priori regions of interest to be selected. TBSS also offers advantages over standard voxel-based analytic techniques by removing the need for spatial smoothing and minimizing the methodological pitfalls caused by misalignment and misregistration, consequently increasing the sensitivity and interpretability of findings.

The present study aimed to use TBSS to compare FA and MD values across PSP, MSA, PD and healthy controls, enabling the characterization of regions of abnormal white matter diffusion properties. We performed a series of analyses to test the hypothesis that white matter changes are present in the superior cerebellar peduncle, superior longitudinal fasciculus and corpus callosum in PSP, middle cerebellar peduncles and motor tracts in MSA, with fewer changes demonstrated in the PD group. To our knowledge this is the first study to assess PD, MSA, PSP and healthy controls in a single study using a whole brain approach.

## Methods

### Participants

Sixteen patients diagnosed with the Richardson’s syndrome variant of PSP, seventeen with MSA and fourteen with PD, according to established criteria, [22]–[24] were recruited successively from the Movement Disorders Clinic at King’s College Hospital and via referrals from clinicians in south east England. Both clinical variants of MSA were included; eleven probable MSA-P (predominant parkinsonian features) and five MSA-C (predominant cerebellar features). All PSP patients were classified as probable, [1] while all patients with PD fulfilled criteria for definite PD [23]. Eighteen healthy age-matched controls were also recruited (spouses and friends of patients) (see Table 1).

**Table 1:** Demographic and clinical data of control subjects and patients with PD, MSA and PSP.

	Control (n = 17)	PD (n = 14)	MSA (n = 16)	PSP (n = 16)	P-value
Age, mean (SD)	63.9 (8.4)	64.7 (6.9)	62.3 (7.3)	69.2 (6.2)	0.056
Sex, M:F	9∶8	7∶7	8∶8	6∶10	NA
Disease duration,mean (SD)	NA	6.6 (2.0)	5.1 (2.7)	5.2 (2.5)	0.181
H&Y* medianscore (range)	NA	2.5 (2.0–3.0)	3.0 (2.5–5.0)	4.0 (3.0–4.0)	<0.001
Schwab and EnglandADL* medianscore (range)	NA	90% (80–100%)	60% (40–80%)	45% (20–80%)	<0.01
UPDRS III,*mean +/− SD (range)	NA	21.8+/−9.6 (5–38)	37.6+/−13.5 (12–62)	35.9+/−6.6 (24–47)	<0.001
Occulomotor Score,†median (range)	NA	0 (0–3)	1.5 (0–5)	16 (7–20)	<0.001
Cerebellar Score,†median (range)	NA	0 (0–2)	7.5 (0–13)	2 (0–6)	<0.05
MMSE, mean (SD)	29 (1)	29.5 (1.1)	28 (2.7)	26 (2.7)	<0.001
DRS, mean (SD)	140 (3.4)	140 (2.9)	135.4 (8.9)	126 (10.2)	<0.001

*For patients taking levodopa drug treatment, scores given in the ‘on’ state.

† From Parkinson’s Plus Scale (Cerebellar Score, maximum 24; Occulomotor Score, maximum 21).

MSA = multiple systems atrophy; PSP = progressive supranuclear palsy; PD = Parkinson’s disease; MSA-P = multiple system atrophy parkinsonian variant; MSA-C = multiple system atrophy cerebellar variant; H&Y = Hoehn and Yahr; ADL = activities of daily living; UPDRS III = Unified Parkinson Disease Rating Scale–part III; MMSE = Mini Mental State Examination; DRS = Mattis Dementia Rating Scale.

### Ethics Statement

The project was approved by research ethics committees of King’s Healthcare NHS Trust and the Institute of Psychiatry and South London and Maudsley NHS Trust. Written informed consent was given by all subjects before participation in the study.

### Clinical and cognitive measures

Within 1 week of the MRI scan each participant was examined by the same clinician (CB). Disease severity was measured using Hoehn and Yahr (H&Y) [25], Schwab and England Activities of Daily Living (ADL) [26] and Unified Parkinson’s Disease Rating Scale Part III (UPDRS-III) [27]. Cerebellar ataxia and occulomotor dysfunction were assessed using the Parkinson’s Plus Scale (PPS) [28]. A higher score on the UPDRS-III (0–108 points) and H&Y (stages 1–5) represents greater impairment, whilst a higher score on the Schwab and England ADL (0–100%) represents less impairment. Global cognitive function was assessed using the Mini-Mental State Examination (MMSE) [29] and Mattis Dementia Rating Scale (DRS) [30].

### Image acquisition

All images were acquired with slices parallel to the anterior commissure–posterior commissure line, on a 1.5-T Signa LX NV/i system (General Electric, Milwaukee, WI), with actively shielded magnetic field gradients (maximum amplitude 40 mTm^−1^). A standard quadrature birdcage head coil was used for both RF transmission and reception.

Using a multislice, peripherally gated echoplanar imaging pulse sequence, each DTI volume was acquired from 60 contiguous 2.5 mm thick slices with field of view (FOV) 240×240 mm and matrix size 96×96, zero-filled to 128×128, giving an in-plane voxel size of 1.875×1.875 mm^2^. Echo time was 107 ms, and effective repetition time was 15 R-R intervals. At each location, 7 images were acquired without diffusion weighting, together with 64 images with a weighting of 1,300 s mm^−2^ applied along directions uniformly distributed in space (Jones et al, 2002). Since acquisition of DTI data were cardiac-gated, scanning time varied according to each subjects pulse rate. For most subjects, scanning time was approximately 25 minutes. A semi-automated EPI quality control procedure was used [31].

### Image processing

An additional visual inspection of diffusion data was further used to exclude data that did not meet quality requirements; one MSA patient and one healthy control subject were excluded due to corrupted scans. Data were then processed using ExploreDTI [32] to correct for the effects of eddy current distortions and head motion. For each subject the b-matrix was then re-oriented to provide a more accurate estimate of tensor orientations. The diffusion-tensor was estimated using a non-linear least square approach, with FA and MD calculated from the diffusion-tensor. Voxel-wise statistical analysis of FA and MD was carried out using TBSS v1.2 (tract-based spatial statistics) (http://www.fmrib.ox.ac.uk/fsl/tbss/) [21], [33], [34] to compare groups. First, each fractional anisotropy image was registered to standard MNI space using the non-linear registration tool in FSL (FNIRT), resulting in a standardised version of each FA image. These steps were repeated for the mean diffusivity data. A voxel-wise average of all subjects was used to create a study-specific mean fractional anisotropy image, which was then ‘skeletonized’ to create a mean fractional anisotropy skeleton, representing the centres of all white matter tracts. To exclude low anisotropic regions in the skeleton, a fractional anisotropy threshold of 0.2 was applied.

### Statistical Analysis

#### Clinical Variables

Clinical variables were analysed using SPSS (version 20); one-way ANOVA with built-in post-hoc Bonferroni tests were used to assess between-group differences in age, duration of disease, UPDRS-III and cognitive measures (MMSE and DRS scores). Post-hoc t-tests were also performed to assess the difference in age between the PSP group and each other group.

A Kruskall-Wallis test was run to determine if there were differences between H&Y, Schwab and England ADL, PPS Occulomotor and Cerebellar scores between clinical groups. Pairwise comparisons were performed using Dunn’s (1964) procedure with a Bonferroni correction for multiple comparisons.

#### Image Analysis

Whole-brain statistical analyses were performed using Randomise v2.1 (FSL). Differences in FA and MD between groups were assessed using a two-sample t test to compare FA and MD values. Clinical groups were first compared with a healthy control population, and then further compared to each other. Additional analyses were run on all subjects as a single group to assess associations between FA and MD values and measures of disease severity (H Schwab and England ADL; UDRS-III), PPS cerebellar and occulomotor scores and cognition (MMSE; DRS).

Age and gender were de-meaned before analysis and used as covariates of no-interest within the voxel-based analysis. Threshold-free cluster enhancement (TFCE) was used in all statistical comparisons to correct for multiple comparisons across space; a non-parametric permutation test was used, in which group membership was permuted 5000 times to generate a null distribution for each contrast. Due to the number of statistical tests carried out an additional Bonferroni correction for multiple comparisons was applied dividing the alpha value by the number of tests each data set was included in. To localize significant voxel effects, contrast maps were subdivided according to the 48 regions of the JHU-ICBM-DTI-81 white matter atlas [35], allowing identification of regions of significance and peak voxels within clusters.

## Results

### Demographic and clinical variables

Diagnostic groups did not differ significantly in age, gender or disease duration in one-way ANOVA analyses. However, post hoc t-tests showed that the PSP group is significantly older than the MSA (p = 0.007) and HC (p = 0.048) groups. The PD group scored lower than the MSA and PSP groups on measures of disease severity; H&Y (p≤0.001), Schwab and England ADL (p≤0.01) and UPDRS III (p≤0.001). The MSA group had more severe cerebellar dysfunction (p≤0.05) than the other two clinical groups, while the PSP group had more severe occulomotor dysfunction (p≤0.001) and greater cognitive impairment; MMSE (p≤0.001) and DRS (p≤0.001) (Table 1).

### PD, MSA and PSP versus healthy controls

First, the white matter maps of each clinical group (PD, MSA, PSP) were compared individually with the maps of age-matched healthy control subjects.

#### PSP

**Figure 1:**
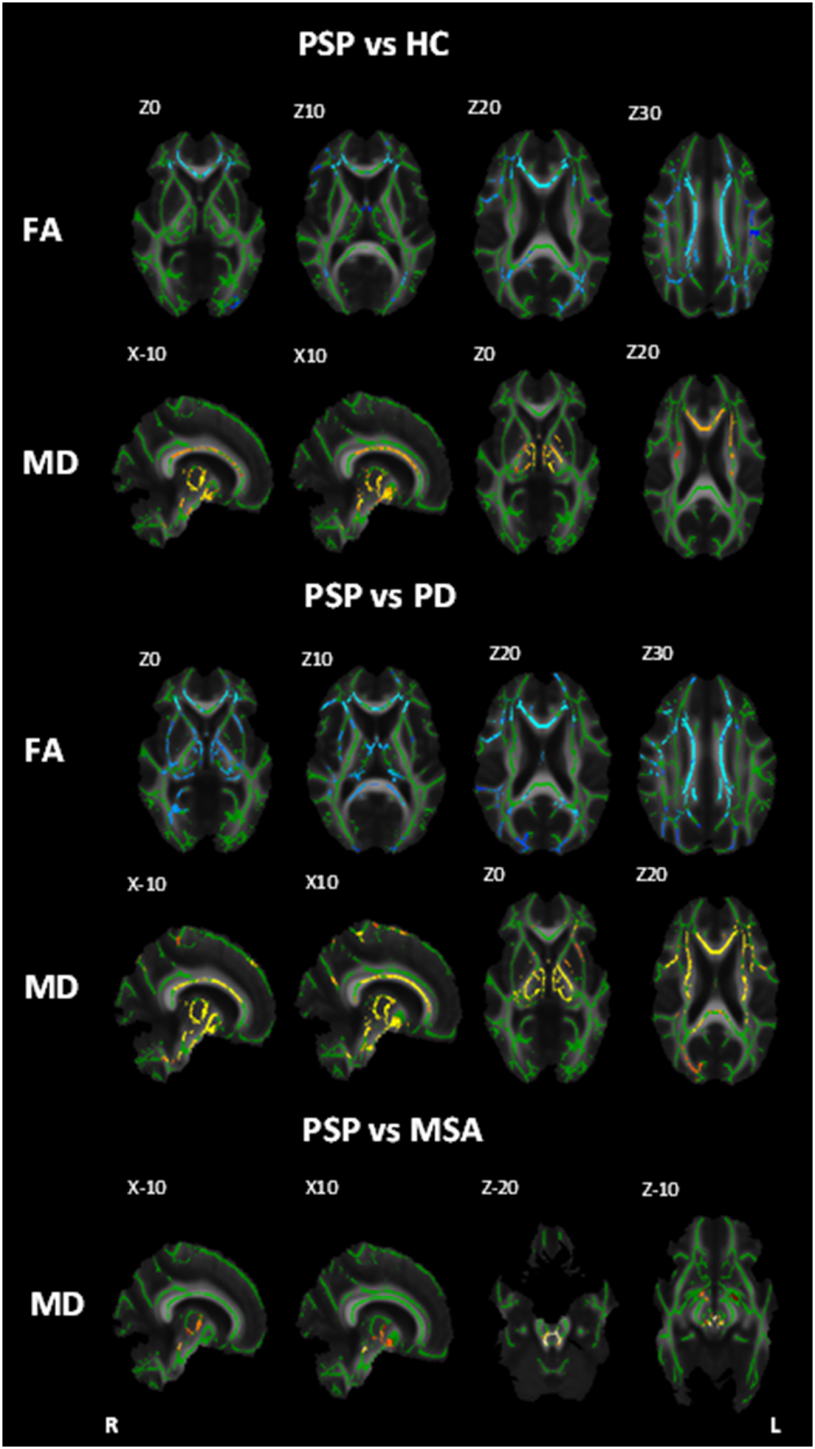
White matter maps showing regions of significant decreased fractional anisotropy and increased mean diffusivity in PSP patients when compared to healthy controls, PD and MSA (Bonferroni corrected alpha = 0.0167). Background image corresponds to the mean fractional anisotropy image of all subjects in standard MNI152 space (radiological view). Fractional anisotropy white matter skeleton is represented by green voxels. Blue voxels represent regions of decreased FA and yellow voxels represent regions of increased MD in the PSP group.

These comparisons revealed regions of reduced FA in PSP in the corpus callosum, anterior and superior corona radiata, superior longitudinal fasciculus/arcuate, and corticospinal tract bilaterally as well as the left posterior corona radiata (Figure 1; Table 2). Regions of increased MD were found in the PSP group in the corpus callosum, superior corona radiata, anterior thalamic radiation, superior cerebellar peduncle, medial lemniscus, cerebral peduncle and posterior limb of the internal capsule bilaterally (Figure 1; Table 3).

**Table 2:** White matter regions of fractional anisotropy changes between PSP, MSA, PD and HC.

Region	Coordinates			PSP<HC	PSP<PD	MSA<PD
	X	Y	Z	P-value	P-value	P-value
**Corpus callosum**						
Genu	4	27	0	0.0008	0.0002	
Body	1	−21	23	0.0006	0.0002	0.0106
Splenium	−18	−47	27	0.0008	0.0002	
**Corona Radiata**						
Left anterior	−17	22	29	0.0011	0.0004	0.0166
Right anterior	18	16	29	0.0006	0.0002	0.0124
Left superior	−18	11	33	0.0008	0.0004	
Right superior	19	12	33	0.0006	0.0002	
Left posterior	−19	−33	33	0.0008	0.0023	
Right posterior	29	−57	20		0.0072	
**Corticospinal**						
Right	20	−31	50	0.0008	0.0006	0.0032
Left	−6	−55	−20	0.0008	0.0092	0.0032
**Longitudinal fasciculus**						
Left superior	−36	−31	34	0.0155		
Right superior	39	−6	30	0.0004	0.0006	
**Thalamic radiation**						
Right anterior	6	−13	14		0.0036	
Left anterior	−4	−14	14		0.0036	0.0096
**Cerebellar peduncle**						
Left superior	−7	−45	−29		0.0038	
Right superior	7	47	−32		0.0038	
Middle	14	−35	−30			0.0021
Left inferior	−11	−46	−30			0.0021
Right inferior	13	−46	−30			0.0021
Pontine crossing tract	2	−31	−30			0.0017
**Lemniscus**						
Left medial	−6	−38	−30			0.0025
Right medial	6	−37	−30			0.0025
**External Capsule**						
Left	−28	14	0		0.0062	
Right	31	12	0		0.0072	
**Internal Capsule**						
Left retrolenticular	−29	−21	0		0.0104	
Right retrolenticular	38	−28	0		0.0068	
Left posterior limb	−20	−14	0		0.0072	0.0077
Right posterior limb	19	−10	0			0.0196
Left anterior limb	−10	5	0		0.0072	0.0098
Right anterior limb	10	5	0		0.0134	
**Thalamic Radiation**						
Right posterior	30	−60	0		0.0068	

All results reported at p<0.05, TFCE and Bonferroni corrected (corrected alpha = 0.0167).

**Table 3:** White matter regions of mean diffusivity changes between PSP, MSA, PD and HC.

Region	Coordinates			HC<PSP	HC<MSA	PD<PSP	PD<MSA	MSA<PSP
	X	Y	Z	P-value	P-value	P-value	P-value	P-value
**Corpus Callosum**								
Body	0	14	20	0.005				
Genu	2	26	10	0.006		0.0002		
Splenium	14	−36	26	0.005		0.0002		
**Corona Radiata**						0.0017		
Left anterior	−26	30	12					
Right anterior	27	26	12			0.0002		
Left superior	−27	7	26	0.0145		0.0008	0.0040	
Right superior	27	9	26	0.0145		0.0002	0.0040	
Left posterior	−26	−32	27			0.0002		
Right posterior	26	−31	27			0.0013		
**Corticospinal**						0.0002		
Left	−10	−23	−24		0.0028		0.0004	
Right	10	−27	−27		0.0017		0.0004	
**Longitudinal fasciculus**								
Left superior	−32	−29	39			0.0006		
Right superior	35	4	27			0.0002		
**Thalamic radiation**								
Left anterior	4	−19	−7	0.0008		0.0002		
Right anterior	−6	−19	−1	0.0013		0.0002		0.0028
**Cerebellar peduncle**								0.0023
Left superior	−3	−29	−19	0.0006		0.0002	0.0025	
Right superior	4	−29	−19	0.0011		0.0002	0.0008	0.0021
Left inferior	−8	−53	−21		0.0015		0.0002	0.0021
Right inferior	8	−53	−21		0.0013		0.0002	
Middle	14	−35	−31		0.0008		0.0002	
Pontine crossing tract	4	−30	−28		0.0015		0.0002	
**Lemniscus**								
Left medial	−5	−35	−26	0.0008	0.0017	0.0002		
Right medial	6	−35	−25	0.0008	0.0008	0.0002		
**Cerebral peduncle**								
Left	−10	−26	−12	0.0013		0.0002	0.0008	
Right	12	−25	−12	0.0013		0.0002	0.0008	
**Internal Capsule**								
Left posterior limb	−10	−8	0	0.0013		0.0002	0.0041	
Right posterior limb	11	−8	0	0.0021		0.0002	0.0041	
Left anterior limb	−10	0	0			0.0002		
Right anterior limb	10	0	0			0.0002		
**External capsule**								
Right	33	−20	0			0.0028	0.0028	
Left	−30	−13	14			0.0028	0.0041	

All results reported at p<0.05, TFCE and Bonferroni corrected (corrected alpha 0.0167).

#### MSA

**Figure 2:**
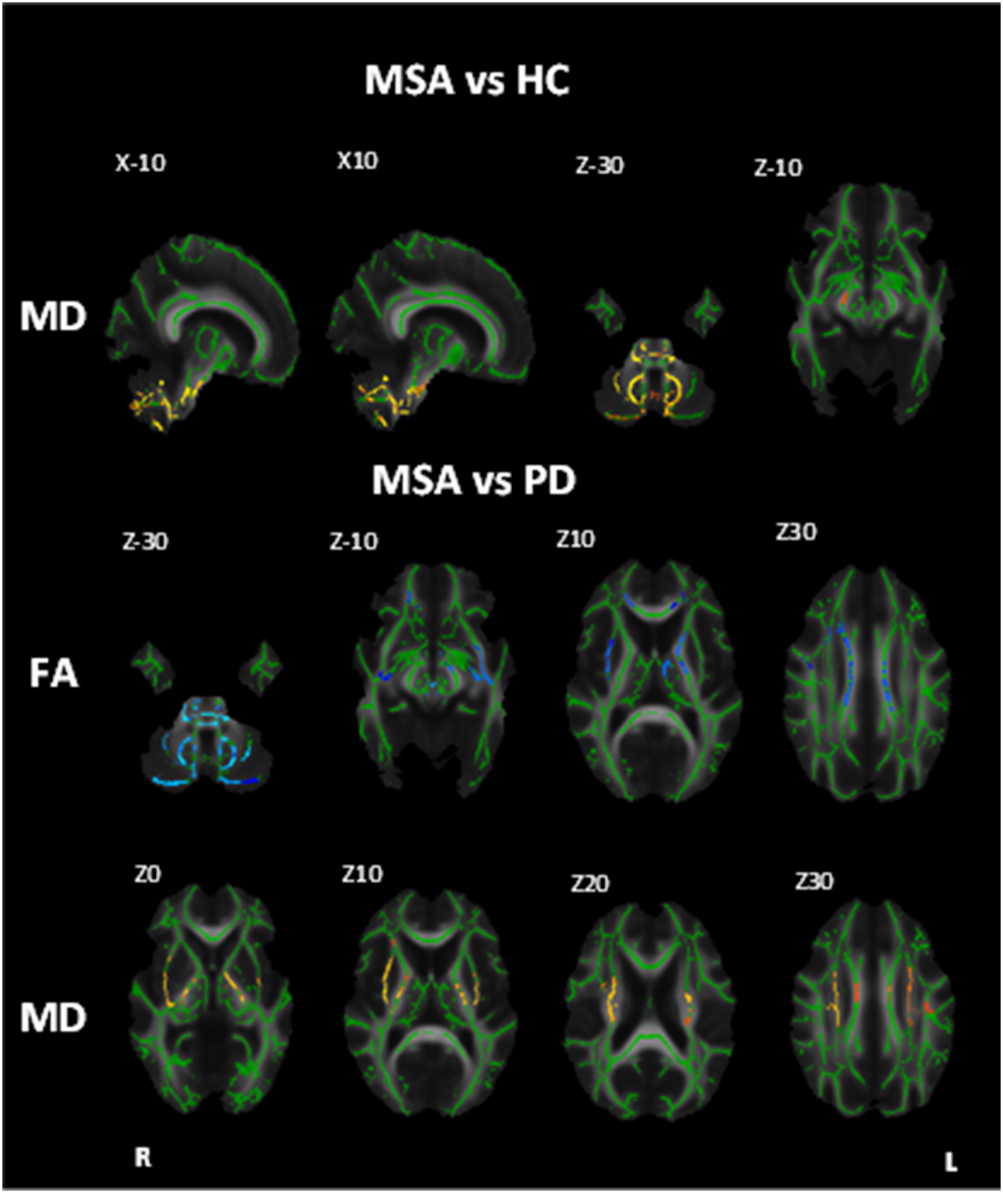
White matter maps showing regions of significant decreased fractional anisotropy and increased mean diffusivity in MSA patients when compared to healthy controls and PD (Bonferroni corrected alpha = 0.0167). Background image corresponds to the mean fractional anisotropy image of all subjects in standard MNI152 space (radiological view). Fractional anisotropy white matter skeleton is represented by green voxels. Blue voxels represent regions of decreased FA and yellow voxels represent regions of increased MD in the PSP group.

MSA patients showed increased MD in the corticospinal tract, middle and inferior cerebellar peduncles, and medial lemniscus (Figure 2; Table 3).

#### PD

There were no significant differences between the PD patients and healthy controls.

All results are Bonferroni corrected (corrected alpha = 0.0167).

### Clinical group comparisons

#### PSP vs PD

Reduced FA was identified in the PSP group when compared to PD in the corpus callosum, corona radiata, corticospinal tract, anterior thalamic radiation, superior cerebellar peduncle, external capsule, retrolenticular and anterior limb of the internal capsule bilaterally, as well as the right superior longitudinal fasciculus, left posterior limb of the internal capsule and the right posterior thalamic radiation (Figure 1; Table 2). Increased MD was also seen in PSP compared to PD, affecting the corpus callosum, corona radiata, superior cerebellar peduncles, anterior thalamic radiation, superior longitudinal fasciculus, medial lemniscus, cerebral peduncle, posterior and anterior limbs of the internal capsule and the external capsule bilaterally (Figure 1; Table 3).

#### MSA vs PD

When compared to PD the MSA group displayed reduced FA in the body of the corpus callosum, anterior corona radiata, corticospinal tract, middle and inferior cerebellar peduncles, medial lemniscus, and posterior limb of the internal capsule bilaterally, as well as the left anterior limb of the internal capsule and left anterior thalamic radiation (Figure 2; Table 2). Increased MD was found in the superior corona radiata, corticospinal tract, superior, middle and inferior cerebellar peduncles, posterior limb of the internal capsule, cerebral peduncle and the external capsule bilaterally (Figure 2; Table 3).

#### PSP vs MSA

The PSP group showed increased MD when compared to MSA in the anterior thalamic radiation and superior cerebellar peduncles (Figure 1; Table 2).

There were no regions in which PD showed significantly reduced FA or decreased MD compared to either MSA or PSP. All results passed bonferroni correction (corrected alpha = 0.0167).

### Relationship between FA and MD values and clinical variables

**Figure 3:**
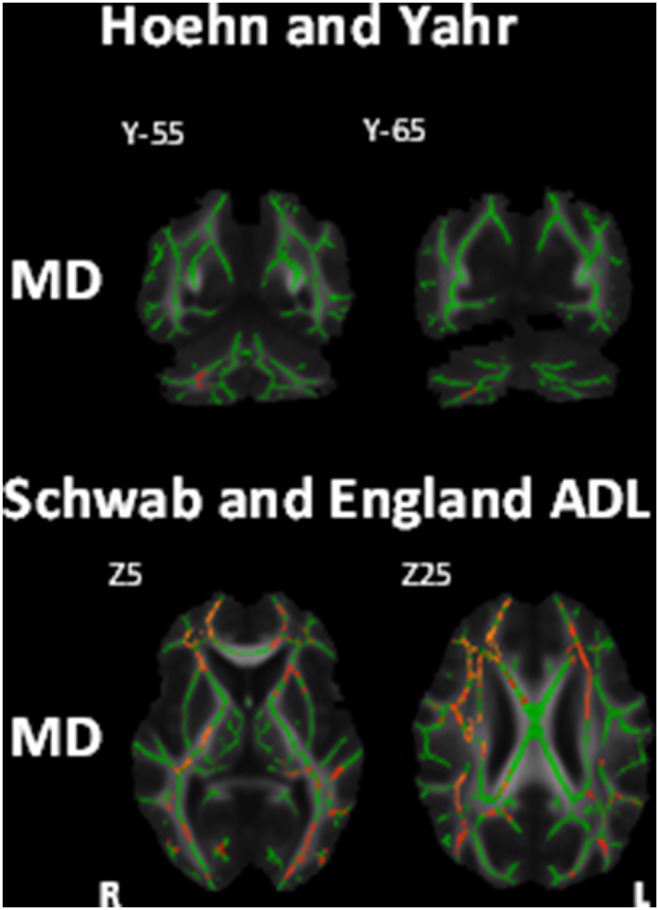
White matter maps showing regions of significant correlation between mean diffusivity and measures of disease severity (Bonferroni corrected alpha = 0.0167). Top row coronal view, bottom row axial view. Background image corresponds to the mean fractional anisotropy image of all subjects in standard MNI152 space (radiological view). Fractional anisotropy white matter skeleton is represented by green voxels. Blue voxels represent regions of decreased FA and yellow voxels represent regions of increased MD in the PSP group.

Measures of disease severity showed significant correlation with MD values after Bonferroni correction. Disease severity as measured by the Schwab and England ADL correlated negatively with MD values in all main white matter tracts that showed changes in this study (see Table 2 and 3) with a right lateralisation of the corona radiata, corpus callosum and superior longitudinal fasciculus (Figure 3). MD values in the middle cerebellar peduncles also correlated positively with scores on the H&Y scale of disease severity (Figure 3).

### Radial and Axial Diffusivity

As requested by one of the reviewers additional analyses were undertaken to assess group differences in radial and axial diffusivity. As these measures were not part of the hypothesis for this study we have included these results (see Table S1 and S2).

## Discussion

To our knowledge this is the first study to investigate DTI indices consistent with white matter microstructural properties using a whole-brain automated technique to directly compare PD, MSA, PSP and healthy control subjects. We demonstrate changes in FA and MD measures that are consistent with white matter tract degeneration in PSP encompassing all of the main white matter tracts including the corpus callosum, superior longitudinal fasciculus, superior cerebellar peduncles and cingulum, in line with recent reports [12]–[17], [36]. These results support the work of Agosta and colleagues, whilst also identifying additional regions that may be implicated in the disease process by using an exploratory whole-brain approach that does not rely on a priori regions of interest. Other regions showing changes in PSP included the corona radiata, thalamic radiation, medial lemniscus, cerebral peduncle, internal capsule and the external capsule. In MSA changes reflecting white matter tract degeneration were observed in the middle and inferior cerebellar peduncle, corticospinal tract, lemniscus, cerebral peduncle, left posterior limb of the internal capsule and left external capsule. Consistent with previous studies, white matter was found to be intact in PD [20], [37]–[39].

Of particular interest is the superior cerebellar peduncles in PSP which showed significant changes that may be reflective of neurodegeneration. In contrast, patients with MSA displayed significant changes in the middle and inferior cerebellar peduncles compared to healthy controls and in the superior, middle and inferior cerebellar peduncles when compared with PD. These results are in line with a previous study that has reported the ability to differentiate between patients with PD, MSA and PSP based on diffusion measures in the middle cerebellar peduncles [9] – a region that appears to be particularly affected in MSA. In this study, MD values across the cerebellar peduncle also correlated with patients H&Y and Schwab and England ADL scores; measures of disease severity, which may mean that degeneration in this region could be used to assess disease progression and that cerebellar peduncle diffusion measures along with a network of other regions of high significance such as the corpus callosum and corona radiata may have the potential to be used to discriminate between MSA and PSP. Furthermore, these results confirm that degeneration of these tracts does not occur in PD.

Interestingly, superior cerebellar peduncles abnormalities in PSP relative to PD, MSA and control subjects were found only for MD value, while reduced FA identified greater changes in the left peduncle relative to PD. This discrepancy between fractional anisotropy and mean diffusivity results could be due to differences in fiber architecture between subjects leading to increased variability of fractional anisotropy across groups – fiber architecture is a major determinant of anisotropy in healthy brains [40]. Mean diffusivity may provide a more direct measure of neuronal integrity because it is believed to be less affected by the fiber architecture or its structural organisation.

Significantly reduced FA and MD were observed in PSP in the genu, body and splenium of the corpus callosum. These results are more widespread than in previous studies that have reported only partial involvement of the corpus callosum [12], [14], [36], [41]. The fibres of the genu of the corpus callosum provide connections to the premotor and supplementary motor areas of the superior frontal gyrus – regions of grey matter that have been previously implicated in the disease [12], [42], [43] whilst the splenium of the corpus callosum communicates somatosensory information between the parietal lobe and visual centre of the occipital lobe. FA and MD values in the corpus callosum did not correlate with any clinical variables. It could be that loss of integrity in these white matter tracts is a result of Wallerian degeneration, a secondary degeneration of axons following cortical grey matter atrophy in regions that have been found to be related to clinical symptoms and severity.

An alternative explanation to Wallerian degeneration could be activated microglia or tau deposition causing changes to the microstructural properties of these white matter tracts. The pattern of degeneration of white matter tracts in PSP seen in this study is consistent with the pattern of activated microglia found in a previous study [44]. The activation of microglia is a reactive response to pathology [45] and so is likely to indicate where pathology exists but not the cause of it. The presence of microglia in white matter tracts has been shown to correlate with tau burden in the cerebral peduncle and internal capsule, but not the superior cerebellar peduncle where microglial presence was higher. Thus in some regions microglia may become active in response to tau related pathology, whilst in others microgliosis may be related to degeneration that is not associated with tau pathology, but another cause of initial degeneration, with microgliosis leading to further tissue pathology [46]. Similarly, in MSA, both activated microglia and glial cytoplasmic inclusion (GCI) are present in affected white matter tracts [44], [47], [48]. Further pathological studies are required to understand the impact of protein dysfunction, microgliosis and Wallerian degeneration on white matter integrity in these populations.

This study additionally identified lateralisation of FA changes in regions including the left posterior corona radiata, right superior longitudinal fasciculus and right posterior thalamic radiation in PSP and in the left anterior thalamic radiation, left anterior limb of the internal capsule in MSA patients. Hemispheric lateralisation has not been well studied in these patient samples and has not been reported in previous studies of this nature, thus these results will need to be verified in a larger sample size to ensure that this lateralisation is not an artefact.

Our study has a number of strengths. This is the first study to explore differences and similarities of DTI indices that are indicative of white matter tract degeneration in PD, MSA and PSP populations in a single study. Secondly, this is the first study to apply a whole-brain automated technique in these populations, avoiding the use of a priori defined ROIs. TBSS offers several other advantages over other methods, for example it removes the need for spatial smoothing and minimises the methodological pitfalls caused by misalignment and misregistration, consequently improving the sensitivity and interpretability of findings. The main limitation of this study is the sample size for each clinical group, the statistical power of the study would be increased with larger group sizes. In addition, results may have been affected by the slightly older age of the PSP patients, thus age was included as a nuisance factor in all analyses to account for this difference. It would also have been beneficial to run a battery of neuropsychological tests on all subjects to rule out confounding effects due to other disorders or conditions. Due to the inclusion of a series of statistical tests with the four groups, a Bonferroni correction was applied which is likely to have reduced the power of the results. Finally, in this study both subtypes MSA-C and MSA-P patients were included as a single MSA group, it is possible that there are subtle pathological differences between these groups, thus in future it would be interesting to look at both of these subtypes as separate groups.

It is clear from this study that in the established disease state PSP patients have profound and widespread white matter alterations. Similarly, MSA patients also display white matter changes in the later stages of the disease. Despite PD patients having longer mean disease duration than MSA and PSP, they scored significantly lower on measures of disease severity and displayed unaffected white matter. This evidence, along with positive correlations between disease severity and white matter changes, provides evidence for slower neurodegeneration along with slower symptom progression [1] in PD compared to other Parkinsonian syndromes. This study provides a comprehensive characterisation of white matter alterations in MSA and PSP that may be used to identify regions of particular interest in future studies assessing early stage disease and may in future aid in the identification of an in vivo biomarker. In conclusion, this work provides evidence for widespread changes in FA and MD that are consistent with white matter neurodegeneration in both MSA and PSP patients, whilst white matter remains preserved in PD. These disruptions are particularly evident in the cerebellar peduncles, supratentorial regions and association fibers.

## Supporting Information

Table S1 **White matter regions of radial diffusivity changes between PSP, MSA, PD and HC.** All results reported at p<0.05, TFCE corrected.
(DOCX)

Table S2 **White matter regions of axial diffusivity changes between PSP, MSA, PD and HC.** All results reported at p<0.05, TFCE corrected.
(DOCX)
