# Supplementary material for: Diffusion Tensor Imaging of Parkinson’s Disease, Multiple System Atrophy and Progressive Supranuclear Palsy: A Tract-Based Spatial Statistics Study
Source: PLoS One. 2014 Nov 18;9(11):e112638. doi: 10.1371/journal.pone.0112638 (PMC4236070; doi:10.1371/journal.pone.0112638)
Supplement: Table S1 — White matter regions of radial diffusivity changes between PSP, MSA, PD and HC. All results reported at p<0.05, TFCE corrected. (DOCX) [file pone.0112638.s001.docx]

| **Region**  **Table 2**:*White matter regions of radial diffusivity changes between PSP, MSA, PD and HC. All results reported at p<0.05, TFCE corrected .* | **Coordinates** | | | **PSP>HC** | **MSA>HC** | **PSP>PD** | **MSA>PD** | **PSP>MSA** |
| --- | --- | --- | --- | --- | --- | --- | --- | --- |
|  | X | Y | Z | P-value | P-value | P-value | P-value |  |
| **Corpus callosum** |  |  |  |  |  |  |  |  |
| Genu | 2 | 26 | 1 | 0.0083 |  | 0.0019 | 0.0264 |  |
| Body | 1 | --8 | 26 | 0.0021 |  | 0.0006 | 0.0134 |  |
| Splenium | 18 | -45 | 32 | 0.0211 |  | 0.0047 |  |  |
| **Corona Radiata** |  |  |  |  |  |  |  |  |
| Left anterior | -18 | 40 | 1 | 0.0023 |  | 0.0013 |  |  |
| Right anterior | 20 | 40 | 1 | 0.0045 |  | 0.0013 | 0.0326 |  |
| Left superior | -18 | 11 | 33 |  |  | 0.0017 |  |  |
| Right superior | 19 | 12 | 33 |  |  | 0.0013 |  |  |
| Left posterior | -19 | -33 | 33 |  |  | 0.0011 |  |  |
| Right posterior | 29 | -57 | 20 |  |  | 0.0087 |  |  |
| **Corticospinal** |  |  |  |  |  |  |  |  |
| Right | 10 | -23 | -24 |  | 0.0375 |  | 0.0004 |  |
| Left | -10 | -23 | -24 |  | 0.0328 |  | 0.0008 |  |
| **Longitudinal fasciculus** |  |  |  |  |  |  |  |  |
| Right superior | 35 | -41 | 32 | 0.0211 |  |  |  |  |
| **Cerebral Peduncle** |  |  |  |  |  |  |  |  |
| Left | -14 | -18 | -15 | 0.0126 | 0.0432 | 0.0041 | 0.0030 |  |
| Right | 14 | -18 | -15 | 0.0483 | 0.0378 | 0.0034 | 0.0051 |  |
| **Cerebellar peduncle** |  |  |  |  |  |  |  |  |
| Left superior | -5 | -34 | -19 | 0.0053 | 0.0053 | 0.0011 |  |  |
| Right superior | 7 | -32 | -19 | 0.0051 | 0.0053 | 0.0019 |  | 0.0398 |
| Left Middle | -25 | -61 | -40 |  | 0.0019 | 0.0177 |  |  |
| Right Middle | 26 | -63 | 40 |  | 0.0008 | 0.0066 | 0.0002 |  |
| Left inferior | -9 | -42 | -33 | 0.0064 | 0.0036 | 0.0011 | 0.0002 |  |
| Right inferior | 9 | -42 | -36 | 0.0085 | 0.0023 | 0.0019 | 0.0006 |  |
| Pontine crossing tract | 0 | -32 | -35 | 0.0070 | 0.0013 | 0.0015 | 0.0002 |  |
| **Lemniscus** |  |  |  |  |  |  | 0.0002 |  |
| Left medial | -5 | -37 | -30 |  | 0.0149 | 0.0011 |  |  |
| Right medial | 6 | -37 | -30 |  | 0.0172 | 0.0011 |  |  |
| **External Capsule** |  |  |  |  |  |  |  |  |
| Left | -30 | 12 | -5 | 0.0158 |  | 0.0041 |  |  |
| Right | 31 | 12 | 0 |  |  | 0.0083 | 0.0051 |  |
| **Internal Capsule** |  |  |  |  |  |  | 0.0202 |  |
| Left retrolenticular | -29 | -21 | 0 |  |  | 0.0027 |  |  |
| Right retrolenticular | 38 | -28 | 0 |  |  | 0.0060 | 0.0187 |  |
| Left posterior limb | -19 | -11 | 1 | 0.0126 |  | 0.0015 | 0.0121 |  |
| Right posterior limb | 20 | -12 | 1 | 0.0334 |  | 0.0034 | 0.0047 |  |
| Left anterior limb | -11 | 77 | 1 | 0.0158 |  | 0.0087 | 0.0153 |  |
| Right anterior limb | 12 | 9 | 1 | 0.0153 |  | 0.0047 |  |  |
| **Cingulum** |  |  |  |  |  |  |  |  |
| Left | -9 | -25 | 34 |  |  | 0.0170 |  |  |
| Right | 10 | -24 | 34 |  |  | 0.0047 |  |  |
| **Uncinate Fasciculus** |  |  |  |  |  |  |  |  |
| Right | 34 | 1 | -12 |  |  |  |  |  |
| **Fornix** | 0 | -8 | 15 | 0.0072 |  |  | 0.0202 |  |
