# Supplementary material for: Diffusion Tensor Imaging of Parkinson’s Disease, Multiple System Atrophy and Progressive Supranuclear Palsy: A Tract-Based Spatial Statistics Study
Source: PLoS One. 2014 Nov 18;9(11):e112638. doi: 10.1371/journal.pone.0112638 (PMC4236070; doi:10.1371/journal.pone.0112638)
Supplement: Table S2 — White matter regions of axial diffusivity changes between PSP, MSA, PD and HC. All results reported at p<0.05, TFCE corrected. (DOCX) [file pone.0112638.s002.docx]

| **Region** | **Coordinates** | | | **PSP>HC** | **MSA>HC** | **MSA>PD** | **PSP>PD** | **PSP<MSA** | **PSP>MSA** |
| --- | --- | --- | --- | --- | --- | --- | --- | --- | --- |
|  | X | Y | Z | P-value | P-value | P-value | P-value | P-value | P-value |
| **Corpus callosum** |  |  |  |  |  |  |  |  |  |
| Genu | 0 | 21 | 16 | 0.0228 |  |  | 0.0096 |  |  |
| Body | 1 | -11 | 25 | 0.0224 |  |  | 0.0064 |  |  |
| **Corona Radiata** |  |  |  |  |  |  |  |  |  |
| Left anterior | -25 | 22 | 25 | 0.0057 |  |  | 0.0023 |  |  |
| Right anterior | 20 | 40 | 1 |  |  |  |  |  |  |
| Left superior | -26 | -1 | 25 | 0.0038 |  |  | 0.0006 |  | 0.0177 |
| Right superior | 27 | 1 | 25 | 0.0460 |  | 0.0456 | 0.0023 |  |  |
| Left posterior | -19 | -33 | 33 |  |  |  | 0.0060 |  |  |
| Right posterior | 29 | -57 | 20 |  |  |  | 0.0060 |  |  |
| **Corticospinal** |  |  |  |  |  |  |  |  |  |
| Right | 10 | -28 | -23 |  | 0.0064 | 0.0036 |  |  |  |
| Left | -10 | -27 | -23 |  | 0.0387 | 0.0283 |  |  |  |
| **Longitudinal fasciculus** |  |  |  |  |  |  |  |  |  |
| Left superior | -37 | -15 | 25 |  |  |  | 0.0138 |  |  |
| Right superior | 35 | -41 | 32 |  |  |  | 0.0138 |  |  |
| **Cerebral Peduncle** |  |  |  |  |  |  |  |  |  |
| Left | -17 | -14 | -7 | 0.0002 | 0.0417 | 0.0434 | 0.0006 |  | 0.0002 |
| Right | 13 | -25 | -17 | 0.0002 | 0.0117 | 0.0241 | 0.0002 |  | 0.0002 |
| **Cerebellar peduncle** |  |  |  |  |  |  |  |  |  |
| Left superior | -5 | -34 | -19 | 0.0004 |  |  | 0.0002 | 0.0245 | 0.0002 |
| Right superior | 7 | -32 | -19 | 0.0004 |  |  | 0.0002 | 0.0038 | 0.0002 |
| Left Middle | -19 | -56 | -31 |  | 0.0032 | 0.0013 |  | 0.0204 |  |
| Right Middle | 19 | -56 | -31 |  | 0.0006 | 0.0004 |  | 0.0003 |  |
| Left inferior | -10 | -45 | -31 | 0.0015 | 0.0021 |  | 0.0008 |  |  |
| Right inferior | 13 | -46 | -31 | 0.0015 | 0.0025 |  |  |  |  |
| Pontine crossing tract | 0 | -32 | -35 | 0.0070 | 0.0013 | 0.0290 |  |  |  |
| **Lemniscus** |  |  |  |  |  |  |  |  |  |
| Left medial | -1 | -37 | -41 | 0.0041 |  |  | 0.0004 |  | 0.0015 |
| Right medial | 3 | -37 | -41 | 0.0015 |  |  | 0.0004 |  | 0.0004 |
| **External Capsule** |  |  |  |  |  |  |  |  |  |
| Left | -30 | 12 | 13 | 0.0089 |  |  |  |  |  |
| **Internal Capsule** |  |  |  |  |  |  |  |  |  |
| Left posterior limb | -16 | -10 | -2 | 0.0002 |  |  | 0.0019 |  | 0.0004 |
| Right posterior limb | 22 | -19 | -2 | 0.0015 |  |  | 0.0045 |  | 0.0002 |
| Left anterior limb | -16 | 7 | 9 | 0.0075 |  |  | 0.0023 |  |  |
| **Fornix** | 0 | -8 | 15 | 0.0057 |  |  | 0.0045 |  | 0.0339 |
